# Study on wild medicinal plant resources and their applied ethnology in multiethnic areas of the Gansu–Ningxia–Inner Mongolia intersection zone

**DOI:** 10.1186/s13002-023-00585-5

**Published:** 2023-05-20

**Authors:** Jian Xie, Chaoqun Luo, Xingwu Yang, Yan Ren, Xingsheng Zhang, Haoran Chen, Yongxia Zhao, Sha Liu, Faming Wu

**Affiliations:** 1 School of Pharmacy, Zunyi Medical University, Zunyi, 563000 China; 2 Sichuan Normal University Hospital, Chengdu, 610068 China; 3 Southwest Minzu University, Chengdu, 610000 China; 4 Agricultural and Rural Bureau of Pingchuan District, Baiyin, 730900 China

**Keywords:** Ethnobotany, Wild plants, Medicinal plants, Moxibustion, Traditional medicine

## Abstract

**Introduction:**

This study conducted an ethnobotanical survey of wild medicinal plants in the multi-ethnic areas of Gansu–Ningxia–Inner Mongolia intersection zone. Traditional knowledge of medicinal plant use in the region was compiled to identify important medicinal plants currently used for treating relevant diseases and to determine species with potential for development.

**Methods:**

Key informant interviews, semi-structured interviews, participatory rural appraisal methods, and ethnobotanical quantitative evaluation were used to investigate and study the traditional knowledge of local residents' use of wild medicinal plants in the region. The relative importance of the referenced plants was assessed, as well as the prominent species widely used in medicinal applications.

**Results:**

The study found that the region has a total of 204 wild medicinal plant resources, belonging to 149 genera of 51 families. Among these resources, a total of 50 commonly used plants were identified (44 of which were herbs, some of which were multi-origin), belonging to 27 families, with the most species found in the Asteraceae family, with 11 species. These herbs are mainly used for preventing and treating colds and nourishing health, followed by treatment of fever, stomach problems, and bleeding. The most frequently used medicinal plant in the region is “Ai”, which includes *Artemisia argyi* Lévl. et Van. and *Artemisia kanashiroi* Kitam. All respondents provided information about the use of this medicinal plant to varying degrees, followed by *Artemisia annua* Linn., *Ephedra sinica* Stapf, *Taraxacum mongolicum* Hand.-Mazz., *Sonchus arvensis* Linn., *Artemisia capillaris* Thunb., among others.

**Conclusion:**

Our investigation gained a wealth of traditional knowledge about the use of wild herbs, using wild herbs, which plays an important role in the lives of local residents. Especially, the herbs and application methods used for treating colds, bleeding, and stomach problems are worthy of further research and development.

**Supplementary Information:**

The online version contains supplementary material available at 10.1186/s13002-023-00585-5.

## Background

Plant resources are the main source of natural medicines [1], and more than 89% of natural medicines used in China come from plant resources [2]. People have accumulated rich knowledge of plant-based prevention and treatment of diseases in the process of fighting against diseases, and this knowledge is an important part of traditional medicine systems worldwide [3]. Different regions, influenced by factors such as climate, ethnicity, and lifestyle, have formed their own distinctive traditional medical knowledge systems [4–6]. In addition to the traditional Chinese medicine system, China also has well-known traditional medicine systems such as Tibetan medicine, Mongolian medicine, and Miao medicine [7]. Each ethnic group has its own unique knowledge system for the application of plant resources. With the development of society and the integration of ethnic groups, traditional knowledge of the utilization of wild plant resources among multiple ethnic groups in some areas has also been exchanged, forming some traditional cultural systems based on locality rather than ethnicity [8].

Ethnobotanical research in the Daqinggou area of Inner Mongolia, Sa et al. [9] found that Mongolian and Han residents of the region exhibited high similarity in their utilization of wild edible plants. Chao et al. [10] conducted ethnobotanical research in Ashhansumu of Wengniute Banner, Inner Mongolia, and collected 183 Mongolian folk names for plants. Zhu et al. [11] investigated medicinal plants used by ethnic groups in Huanxian County, Gansu. These studies have proven to be of great significance for the development of ethnobotany in semi-arid regions, promoting bioprospecting activities aimed at exploring the biodiversity and cultural diversity and their values to the livelihood and health of residents in ethnic regions, seeking plant resources with economic and social value.

Multiethnic integration has allowed local residents to form a unique traditional knowledge system for the utilization of wild plant resources [8, 12]. On the other hand, due to the low precipitation and large evaporation in this area [13], plant resources are scarce in both species and reserves; this scarcity has led to an extremely high degree of exploration of the wild plant resources in this area, which are widely and fully utilized as food, medicine, feed and fuel [8, 14, 15].

In our previous research, we systematically studied the traditional knowledge of edible plant resources in the region and discovered a rich variety of wild edible plant resources and their utilization methods [8]. However, there has been limited ethnobotanical research conducted on traditional medicinal plants in this region, with only a few studies by Zhang et al. [16] and Li et al. [17].

With the advancement of urbanization, modern agriculture, and medical technology, a large amount of traditional knowledge related to ethnic medicine and local medicine has been lost [8, 18, 19]. The purpose of our study is to explore, record, summarize, and compile the traditional knowledge of using wild medicinal plant resources among the residents in the multi-ethnic regions where Gansu, Ningxia, and Inner Mongolia meet. We aim to document and widely disseminate these traditional knowledge that is facing extinction, which has positive significance for the protection of traditional culture and the sustainable development and utilization of wild medicinal plant resources.

## Materials and methods

### Study area

**Fig. 1:**
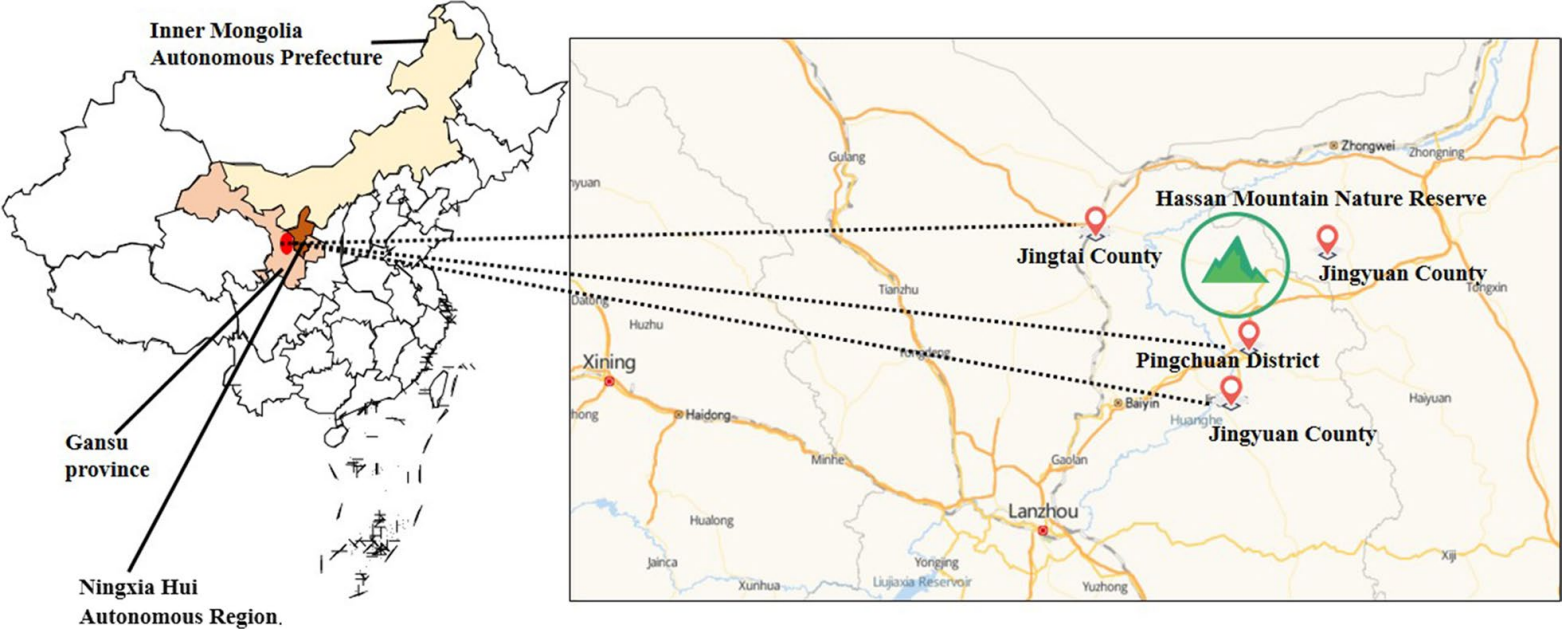
Survey area. Jingyuan County, Pingchuan District and Jingtai County

The study was conducted in the eastern region of Baiyin City, which borders Ningxia and Inner Mongolia, including Jingyuan County, Pingchuan District, and Jingtai County (Fig. 1). This area spans from 36°N to 37°38' N latitude and 103°33' E to 105°51' E longitude, situated at the transition zone of the Loess Plateau, the eastern extension of the Qilian Mountains, and the Tengger Desert, belonging to the transition zone from the temperate semi-arid to arid zone. The average annual temperature ranges from 6 to 9 °C, and the annual precipitation ranges from 180 to 450 mm [20]. Due to the arid climate, the vegetation resources are relatively scarce, mainly consisting of desert and grassland vegetation, with a small amount of forests, shrubs, and wetland vegetation. However, some special medicinal plant resources still exist in this environment (Table 1).

**Table 1:** Basic information of the study areas

County	Location	Population	Main ethnic groups	Main language	GDP/person	Investigation site	GPS
Jingyuan	E 104°13′–105°15′; N 36°–37°15′	373,000	Han Hui Mongolian Tibetan	Chinese	¥22,410	Wanxian Village, Shahe Village, Yongxin Township	E 104°35′, N 37°3′
						Yongxin Township Hassanshan Nature Reserve	E 104°39′, N 37°4′
						Damiao Village	E 104°29′, N 37°12′
						North TanLugou Village	E 104°51′, N 37°12′
						Wulan Town	E 104°41′, N 36°33′
Pingchuan	E 104°18′–105°26′; N 36°10′-37°00′	199,600	Han Hui Tibetan Mongolia	Chinese	¥36,684	Liushui Village, Shuiquan Town	E 104°39′, N 36°54′
						Wangjiashan Town	E 104°49′, N 36°55′
Jingtai	E 103°33′–104°43′; N 36°43′–37°38′	238,000	Han Hui Mongolian	Chinese	¥26,009	Wufo Xingshui Village	E 104°4′, N 37°11′
						Luyangshicheng Village	E 104°9′, N 37°8′

The Hassan Mountain area is the intersection of Gansu, Inner Mongolia and Ningxia [17], and "Hassan" means "jade" in Mongolian. In the history of China, this area is a typical place where nomadic people and farming people coexist together. This area has a long history dating back to the Western Zhou period. During the Han Dynasty, it became an important channel of the Silk Road and a crucial military stronghold in ancient Northwestern China. Over thousands of years, it has formed a multi-ethnic settlement area, mainly inhabited by Han, followed by Hui, Mongolian, Tibetan, Manchu, and other ethnic minorities [21]. The long-term convergence and fusion of multiple ethnic groups have formed unique local cultures and rich traditional knowledge. The economic development level of this region is relatively low, with a small population flow and a severe aging problem. Due to inconvenient transportation, modern medicine is difficult to penetrate into remote villages. Therefore, the local residents plant some medicinal plants, mainly including *Lycium barbarum* Linn. (*L. barbarum* Linn.), *Codonopsis pilosula* (Franch.) Nannf. (*C. pilosula* (Franch.) Nannf.), *Astragalus membranaceus* (Fisch.) Bge. (*A. membranaceus* (Fisch.) Bge.), *Carthamus tinctorius* Linn. (*C. tinctorius* Linn.), and so on, to meet their medical needs [22–24].

### Ethnobotanical information collection

We investigated the medicinal plant resources in this area along the route with provincial roads as the main trunk and rural roads as the branches and focused on the nature reserve (Hassan Nature Reserve). Door-to-door visits were made to attempt to identify local people with specialized knowledge of medicinal plants and record the place names, local names and scientific names of medicinal plants discovered for the first time during the investigation.

To identify the local people with specialized knowledge of medicinal plants, we used key informant interviews, semi-structured interviews, and Participatory Rural Appraisal (PRA) methods. Key informant interviews were conducted with individuals who had traditional knowledge and experience of medicinal plants, such as barefoot doctors and professional collectors [25]. Semi-structured interviews involved using a predetermined list of questions to gather qualitative data (Additional file 1: Table S1) [26]. PRA is a widely used survey method in ethnobotanical research, which involves a dialogue between researchers and local experts, such as doctors and farmers, to assess the overall situation and identify issues related to medicinal plants in the local area [27].

All audio recordings and questionnaires were stored at the Pharmacognosy Teaching and Research Section of the School of Pharmacy, Zunyi Medical University. This study provides important information on the ethnobotanical resources and traditional knowledge of medicinal plants in the study area, which could be useful for the conservation and sustainable utilization of these valuable resources.

The interviews were conducted with the "5W + 1H" method [28] to understand the local residents' traditional knowledge of using wild plants and the participatory observation method [29] was used to understand the methods of collecting and using wild medicinal plants, as well as the types of diseases they treat, their local names, medicinal parts, processing methods, usage methods, and other health care functions. All information was recorded, sorted and analyzed. Before the interview, the objective of the research was explained, and the interviewee was asked to participate in the research and sign the informed consent form to ensure the accuracy of the information provided. In addition, the socioeconomic data of the respondents were recorded, including name, gender, age, education level, occupation and birthplace.

### Quantitative evaluation method of ethnobotanical resources

The national plant cultural significance index (NCSI) was used to evaluate the wild medicinal plants in this area, which was calculated as follows:

$${\text{NCSI}} = {\text{FQI}} \times {\text{AI}} \times {\text{FUI}} \times {\text{PUI}} \times {\text{MFI}} \times {\text{CEI}} \times {\text{DSI}} \times 10^{ - 2}$$

where FQI is the frequency of quotation index, AI is the availability index, FUI is the frequency of utilization index, PUI is the parts used index, MFI is the multifunctional use index, CEI is the curative effect index, and DSI is the drug safety index [30].

The indexes were set according to "Common Research Methods in Ethnobotany" [29] and graded and assigned as follows: the frequency of quotation index (FQI) is the number of people who mentioned a plant among all information reporters; the availability index (AI) is divided into very common (4.0), common (3.0), general (2.0) and uncommon (1.0); the frequency of utilization index (FUI) is divided into more than 10 times a year (5.0), 6–10 times a year (4.0), 2–5 times a year (3.0), at least once a year (2.0), once every 2–3 years (1.0) and not used in the last 5 years (0.5); the parts used index (PUI) is divided into whole plant (5.0), aboveground or underground parts (4.0), stems, leaves, flowers, fruits and seeds (3.0), skins and seeds (2.0), special parts and processed products (1.0); the multifunctional use index (MFI) has a base number of 0, and each additional use adds a natural number where only one use is (1), and five uses are (5); the curative effect index (CEI) is divided into excellent (5.0), very good (4.0), good (3), fair (2) and poor (1); and the drug safety index (DSI) is divided into very high (medicinal and edible: 5.0), high (safe and nontoxic side effects: 4.0), moderately high (with certain side effects: 3.0), moderate (with small toxicity: 2.0) and low (highly toxic: 1.0).

### Specimen identification

We identified species, prepared specimens and filled in the field records of each species and genus for the plants collected with reference to Flora Reipublicae Popularis Sinicae (http://www.iplant.cn/frps) [31], Desert Plants in China [32], Field Guide to Wild Plants of China-Qilian Mountains [33] and Flowering Plants of Hengduan Mountains [34]. The following information was included: collector’s name, scientific name, local name, plant family, sex, flower color and fruit color, as well as other characteristics. The collected information was sorted and analyzed according to the research purpose. The voucher specimens are kept in the Life Science Museum of Zunyi University (wax leaf specimens) and the specimen room (bottled specimens) of the Pharmacognosy Teaching and Research Section of the School of Pharmacy, Zunyi Medical University.

## Results

### Diversity of wild medicinal plant resources

Based on the collated results, a total of 204 species of wild medicinal plants were investigated, belonging to 149 genera in 51 families. Among them, ferns accounted for 1.96%, 0.67%, and 0.98% of the total number of plant families, genera, and species, respectively, with one family, one genus, and two species identified. Gymnosperms accounted for 5.88%, 2.01%, and 2.45% of the total number of plant families, genera, and species, respectively, with three families, three genera, and five species identified. Representative medicinal plants include *Ephedra sinica* Stapf (*E. sinica* Stapf) and *Platycladus orientalis* (Linn.) Franco. (*P. orientalis* (Linn.) Franco.) Monocotyledons accounted for 7.84%, 8.05%, and 7.35% of the total number of plant families, genera, and species, respectively, with four families, twelve genera, and fifteen species identified. Representative medicinal plants include *Iris tenuifolia* Pall. (*I. tenuifolia* Pall.), *Polygonatum cirrhifolium* (Wall.) Royle (*P. cirrhifolium* (Wall.) Royle), and *Polygonatum odoratum* (Mill.) Druce. (*P. odoratum* (Mill.) Druce.) Dicotyledons accounted for 84.31%, 89.26%, and 89.22% of the total number of plant families, genera, and species, respectively, with forty-three families, 133 genera, and 182 species identified. Representative medicinal plants include *Glycyrrhiza uralensis* Fisch. (*G. uralensis* Fisch.), *Polygala sibirica* Linn. (*P. sibirica* Linn.), *Adenophora ningxianica* Hong (*A. ningxianica* Hong), *Cistanche tubulosa* (Schenk) Wight (*C. tubulosa* (Schenk) Wight), *Cynomorium songaricum* Rupr. (*C. songaricum* Rupr.), *Arctium lappa* Linn. (*A. lappa* Linn.), *Hyoscyamus niger* Linn. (*H. niger* Linn.), and *Gentiana dahurica* Fisch. (*G. dahurica* Fisch.)

Single-species families, oligo-species families (containing 2–10 species), and intermediate families (containing 11–20 species) accounted for a considerable proportion of the medicinal plant families in the region. These three categories together accounted for 98.04% of the total number of medicinal plant families surveyed, with 50 families containing 130 genera and 179 species, accounting for 87.25% of the total number of medicinal plant genera and 87.75% of the total number of medicinal plant species. Only one family with more than 20 species was identified, accounting for 1.96% of the total number of plant families, with 19 genera and 25 species identified, accounting for 12.75% of the total number of plant genera and 12.25% of the total number of plant species (Table 2). This indicates that the medicinal plant species in the region tend to be concentrated in a limited number of families, and the phenomenon of dominant families in the region is evident. The distribution of genera also exhibits the same pattern, with all being single-genera or oligogenera, with Potentilla (5 species) and Artemisia (5 species) being the two genera with the most species, containing a total of 10 medicinal plants, accounting for 4.90% of the total number of medicinal plants.

**Table 2:** Cataloguing table of wild medicinal plants in the multi-ethnic areas of Gansu–Ningxia–Inner Mongolia intersection

Family name	Species	Local name	Use part	Processing method	Treatment Functions	Toxic or not	Method of application	Whether included in Chinese Pharmacopoeia	Whether the original is consistent with the Chinese Pharmacopoeia	Voucher numbers
Polygalaceae	*P. sibirica* Linn	Zi-Ru	Velamen	Extract the wood core and dry it after the mallet is flattened	Treat upset and insomnia	No	Boil in water and drink	Yes	No	HS-202208003
Urticaceae	*Urtica fissa* E. Pritz	Xian-Ma	Root	Slice after drying	Treatment of rheumatic diseases	No	Boil in water and drink	No	No	HS-202208007
Berberidaceae	*Radix Berberidis*	Huang-XuanCi	Skin	Peeling	Relieve inflammation/Treat mouth ulcer and gingival inflammation	No	Peel a small piece of stem skin directly and chew it in your mouth	Yes	Yes	HS-202208018
Unknown	Unknown	Bian-Bai	Root	Cut into pieces and dried	strengthen yang-qi/Promote sexual function	Unknown	Soak in wine	–		–
	Unknown	Xin-BuGan	Stalk	Remove leaves	Treatment of hemorrhoids	Unknown	Decoction of fresh stalks for internal use	–		–
Cynomoriaceae	*C. songaricum* Rupr	Suo-Yang	Total plant	Dried in the sun	strengthen yang-qi/Promote sexual function	No	Infusion of wine for internal use/for stewing meat	Yes	Yes	DS-202208017
Brassicaceae	*Lepidium apetalum* Willdenow	La-LaZi	Seed	Dried in the sun	Treating edema	No	Boil in water and drink	Yes	Yes	SH-202208007
	*Isatis indigotica* Fortune	Ban-LanGen	Leave	Fresh product mashed	Treating mumps	No	Fresh and tender leaves are pounded and applied externally	Yes	Yes	SH-202208001
Umbelliferae	*B. smithii* Wolff var. *parvifolium* Shan et Y.Li	Xiao-ChaiHu	Total plant	Dried in the sun	Treating colds	No	Boil in water and drink	No	No	HS-202208021
	*N. forbesii* de Boiss	Qiang-Huo	Root	Dried in the sun	Treating colds, rheumatism	No	Boil in water and drink	Yes	Yes	HS-202208011
Thymelaeaceae	*Stellera chamaejasme* L	Gou-ZhuaZi	Root	Dried in the sun	Insecticidal, anti-itch/Treating various kinds of ringworm, carbuncles and furuncles	Great toxicity	Its powder is used externally	No	No	SH-202208005
Solanaceae	*L. chinense* Miller	Gou-Ci	Fruit	Dried in the sun	Nourishing	No	Direct consumption	Yes	No	SH-202208002
	*H. niger* Linn	Tian-XianZi	Root	Dried in the sun	Treating toothache	Great toxicity	Smoke and fumigate	Yes	Yes	HS-202208014
Ranunculaceae	*A. brachypodum* Diels	Tie-BangChui	Root tuber	Dried in the sun	Dispelling wind and removing dampness/ Treating rheumatism	Great toxicity	Infusion of liquor for external use	No	No	HS-202208015
Lycoperdaceae	*L. seu* Calvatia	Ma-PiPao	Fruiting body	Drying	Traumatic bleeding	No	Direct external application	Yes	Yes	HS-202209001(B)
Ephedraceae	*Ephedra intermedia* Schrenk ex Mey	Ma-Huang	Herbaceous stalk	Cut into pieces and dry in the shade(could not be done in the sun)	Sweating/Treating colds	No	Often decoct with Artemisia annua and dandelion for oral use/scrub	Yes	Yes	HS-202208019
	*E. sinica* Stapf							Yes	Yes	HS-202208024
	*Ephedra equisetina* Bunge							Yes	Yes	HS-202208025
Gentianaceae	*G. dahurica* Fisch	Qin-Jiao	Root	Dried in the sun/Fresh use	Fresh product for the treatment of mumps	No	Fresh product pounded and applied externally	No	No	HS-202208026
Orobanchaceae	*B. rossica* (Chamisso et Schlechtendal) B. Fedtschenko	Da-Yun	Total plant	Dried in the sun	strengthen yang-qi/Promote sexual function	No	Steeped in wine and taken internally	No	No	
	*C. tubulosa* (Schenk) Wight							Yes	Yes	
Polygonaceae	*R. undulatum* Linn	Da-Huang	Root	Slice dried	Laxative, fire cleansing/treatment of mouth ulcers, bad breath, food accumulation	No	Decoction of water for internal use	No	No	HS-202208027
	*R. franzenbachii* Munt							No	No	HS-202208028
Chenopodiaceae	*Kochia scoparia* (Linn.) Schrad	Qian-SaoZhou	Fruit/Stems and leaves	Dried in the sun	Itchy skin	No	Decocted in water for bathing	Yes	Yes	SH-202208003
Compositae	*A. kanashiroi* Kitam	HanAi	Blade/Total plant	Dry in the shade, take leaves and pound them	Warming the meridians/abdominal pain, headache, rheumatism, pediatric fright, prolapsed uterus, postpartum dew, menstrual disorders, etc	No	Ai moxibustion/Leaves or whole plant decoction for internal use or bathing	No	No	YX-202208001
	*A. argyi* Lévl. et Van	ShuiAi	Blade/Total plant	Dry in the shade, take leaves and pound them	Warming the meridians/abdominal pain, headache, rheumatism, pediatric fright, prolapsed uterus, postpartum dew, menstrual disorders, etc	No	Ai moxibustion/Leaves or whole plant decoction for internal use or bathing	Yes	Yes	YX-202208002
	*A. annua* Linn	Huang-Hao	Aboveground parts	Dried in the sun/Be placed in the shade to dry	Treating colds	No	Decoction of water for internal use/Scrubbing	Yes	Yes	SH-202208018
	*T. mongolicum* Hand.-Mazz	Huang-ErCai	Total plant	Dried in the sun	Bring down a fever/Treating colds、mumps	No	Decoction of water for internal use/Scrubbing	Yes	Yes	SH-202208012
	*Artemisia capillaris* Thunb. (*A. capillaris* Thunb.)	Yin-Chen	Total plant	Dried in the sun	Treating colds	No		Yes	Yes	SH-202208010
	*Cephalanoplos segetum* Bge.Kitam	Ci-JiaGai	Stems and leaves	Fresh product mashed	Hemostasis/Traumatic bleeding	No	Fresh stems and leaves pounded and applied externally	Yes	No	SH-202208022
	*Cirsium japonicum* DC	Da-CiJiaGai	Stems and leaves	Fresh product mashed	Hemostasis/Traumatic bleeding	No	Fresh stems and leaves pounded and applied externally	Yes	Yes	SH-202208033
	*S. arvensis* Linn	Tian-KuCai	Total plant	Dried in the sun	Treating gastric disease/gastric ulcer/cancer	No	Boil in water and drink	No	No	YX-202208003
	*M. tataricum* (Linn.) DC	Ma-KuCai	Total plant	Dried in the sun	Treating gastric disease/gastric ulcer/cancer	No	Boil in water and drink	No	No	YX-202208004
	*A. lappa* Linn	Da-NiuCai	Root	Dried in the sun	Treating wind-heat colds and toothaches	No	Boil in water and drink	No	No	HS-202208029
	*Xanthium sibiricum* Patrin ex Widder	Cang-Er	Fruit	Dried in the sun	Treating the rhinitis/Itchy skin	Minor toxicity	Oil drip nose after frying/Decocted in water for bathing	Yes	Yes	DS-202208004
Leguminosae	*A. chrysopterus* Bunge	Huang-Qi	Root	Dried in the sun	Tonifying Qi/treating various deficiencies	No	Boil in water and drink	No	No	HS-202208033
	*G. uralensis* Fisch	Gan-Cao	Root	Cut into pieces and dried in the sun	Treating cough	No	Boil in water and drink	Yes	Yes	SH-202208009
Euphorbiaceae	*E. esula* Linn	Mao-ErYan	Milk	Fresh use	Insecticidal, anti-itch/Treating various kinds of ringworm, carbuncles and furuncles	Moderate toxicity	After breaking the fresh stem, apply its latex to the affected area	No	No	SH-202208017
Labiatae	*Thymus mongolicus* Ronn	Di-Jiao	Aboveground parts	Be placed in the shade to dry	Aiding digestion	No	Add to food after crushing	No	No	HS-202208030
Cupressaceae	*P. orientalis* (Linn.) Franco	Bai-Shu	Twig	Fresh use/be placed in the shade to dry	Treating nosebleeds	No	Freshly mashed and stuffed nostrils/Boil in water and drink	Yes	Yes	YX-202208010
	*Sabina vulgaris* Ant	Di-Bai	Stem	Fresh use/Be placed in the shade to dry	Preservative/Disinfection and sterilization germs	No	The coffin of the dead is filled with stems and branches of herbs to have the effect of embalming; dried stems and branches of herbs burned and smoked to have the effect of extermination	No	No	HS-202208023
Tamaricaceae	*Tamarix karelinii* Bunge	Gui-Liu	Twig	Dried in the sun	Itchy skin	No	Decoct with water for bathing	No	No	YX-202208017
Plantaginaceae	*Plantago minuta* Pall	Che-QianCao	Whole grass	Fresh use/Dried in the sun	Irregular urination	No	Fresh product pounded and juiced to drink or dried product decocted in water to drink	No	No	WX-202208001
	*Plantago depressa* Willd							Yes	Yes	WX-202208002
	*Plantago major* Linn							No	No	WX-202208003
Rosaceae	*Armeniaca vulgaris* Lam. var. *ansu* (Maxim.) Yü et Lu	Xing-He	Seed kernel	Dried in the sun	Cough-cold	No	Boil in water and drink	No	No	HS-202208044(B)
Liliaceae	*Lilium pumilum* DC	San-DanHua	Bulb	Dried in the sun	Nourishing	No	Boil in water and drink or make soup	No	No	HS-202208042
Moraceae	*Cannabis sativa* Linn	Ma-ZiYou	Seed	Extract oil	Constipation	No	Eat with meal or drink in small quantities	Yes	Yes	YX-202208022
Zygophyllaceae	*Tribulus terrester* Linn	Ba-JiaoZi	Fruit	Dried in the sun	Itchy skin	No	Often used with kochiae fructus, decoct with water for bathing	Yes	Yes	SH-202208021
Labiatae	*S. baicalensis* Georgi	Huang-Qin	Root	Dried in the sun	Treat all kinds of excessive internal heat, and also treat wind-heat cold	No	Boil in water and drink	Yes	Yes	SH-202208004

Based on fieldwork and interviews with 105 informants, we classified the medicinal plants into several categories based on their usage parts, including whole plant, root and rhizome, stem and leaf, flower, fruit, and bark. The root and rhizome category has the most medicinal plants, including *G. dahurica* Fisch., *Bupleurum smithii* Wolff var. *parvifolium* Shan et Y.Li (*B. smithii* Wolff var. *parvifolium* Shan et Y.Li), *P. sibirica* Linn., *Notopterygium forbesii* de Boiss. (*N. forbesii* de Boiss.), *Rheum undulatum* Linn. (*R. undulatum* Linn.), and *Cimicifuga foetida* Linn. (*C. foetida* Linn.) The whole plant category includes *Artemisia annua* Linn. (*A. annua* Linn.), *Taraxacum mongolicum* Hand.-Mazz. (*T. mongolicum* Hand.-Mazz). The fruit category has *Ziziphus jujuba* Mill. var. *spinosa* (Bunge) Hu ex H.F.Chow. (*Z. jujuba* Mill. var. *spinosa* (Bunge) Hu ex H.F.Chow.), *Hippophae rhamnoides* Linn. (*H. rhamnoides* Linn.) The flower category includes *C. tinctorius* Linn., and the bark category includes *Acanthopanax giraldii* Harms (*A. giraldii* Harms), *Lycium chinense* Miller (*L. chinense* Miller) (Lycii Cortex), etc. [8].

### Basic information of ethnology information respondents

**Fig. 2:**
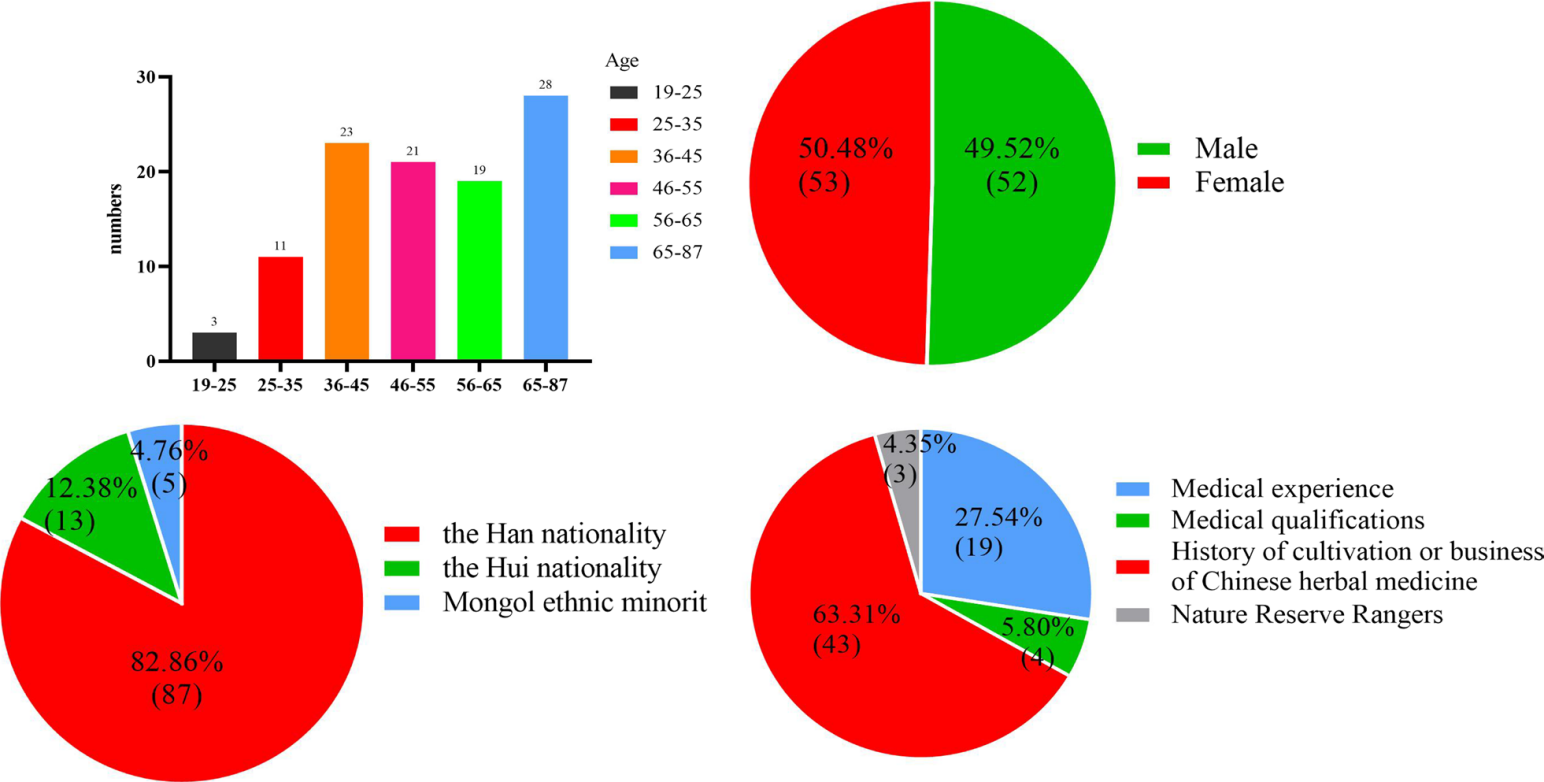
Basic information about the interviewees

In addition, we also investigated the basic information of the informants, including their age, gender, ethnicity, and occupation. The results showed that the age of the 105 informants ranged from 19 to 87 years old, with 3 people under 25 years old, 11 people between 25 and 35 years old, 23 people between 36 and 45 years old, 21 people between 46 and 55 years old, 19 people between 56 and 65 years old, and 28 people over 65 years old. Among them, 53 were male and 52 were female, with a gender ratio of nearly 1:1. There were 87 Han informants, 13 Hui informants, and 5 Mongolian informants. Among the 105 informants, 23 had medical experience (including 4 with medical qualifications), 43 had experience in cultivating or operating Chinese herbal medicine, and 3 were forest rangers in natural reserves (Fig. 2).

### Utilization of wild medicinal plants in the Hassan Mountain area

A total of 105 respondents provided information on the use of 44 medicinal plants, which involved 50 wild medicinal plant species (some of which have multiple sources), representing only one-fourth of the 204 medicinal plant species discovered during our investigation. These 50 wild medicinal plants traditionally used by local residents belong to 27 families, including one species of fungi (*Lasiosphaera seu* Calvatia (*L. seu* Calvatia)) and three species of Ephedra (Ephedraceae), with *E. sinica* Stapf being the most widely used. Of the 46 species from 25 families of angiosperms, the Compositae family has the most species, with 11 species accounting for 22% of the total number. Among them, *E. sinica* Stapf, *Artemisia argyi* Lévl. et Van. (*A. argyi* Lévl. et Van.)/*Artemisia kanashiroi* Kitam. (*A. kanashiroi* Kitam.) (uncertain), *A. annua* Linn., and *T. mongolicum* Hand.-Mazz. are the most representative wild medicinal plants in the area. In addition, two medicinal plants (Bian-Bai and Xin-BuGan) were not identified with certainty due to lack of corresponding plant species.

**Fig. 3:**
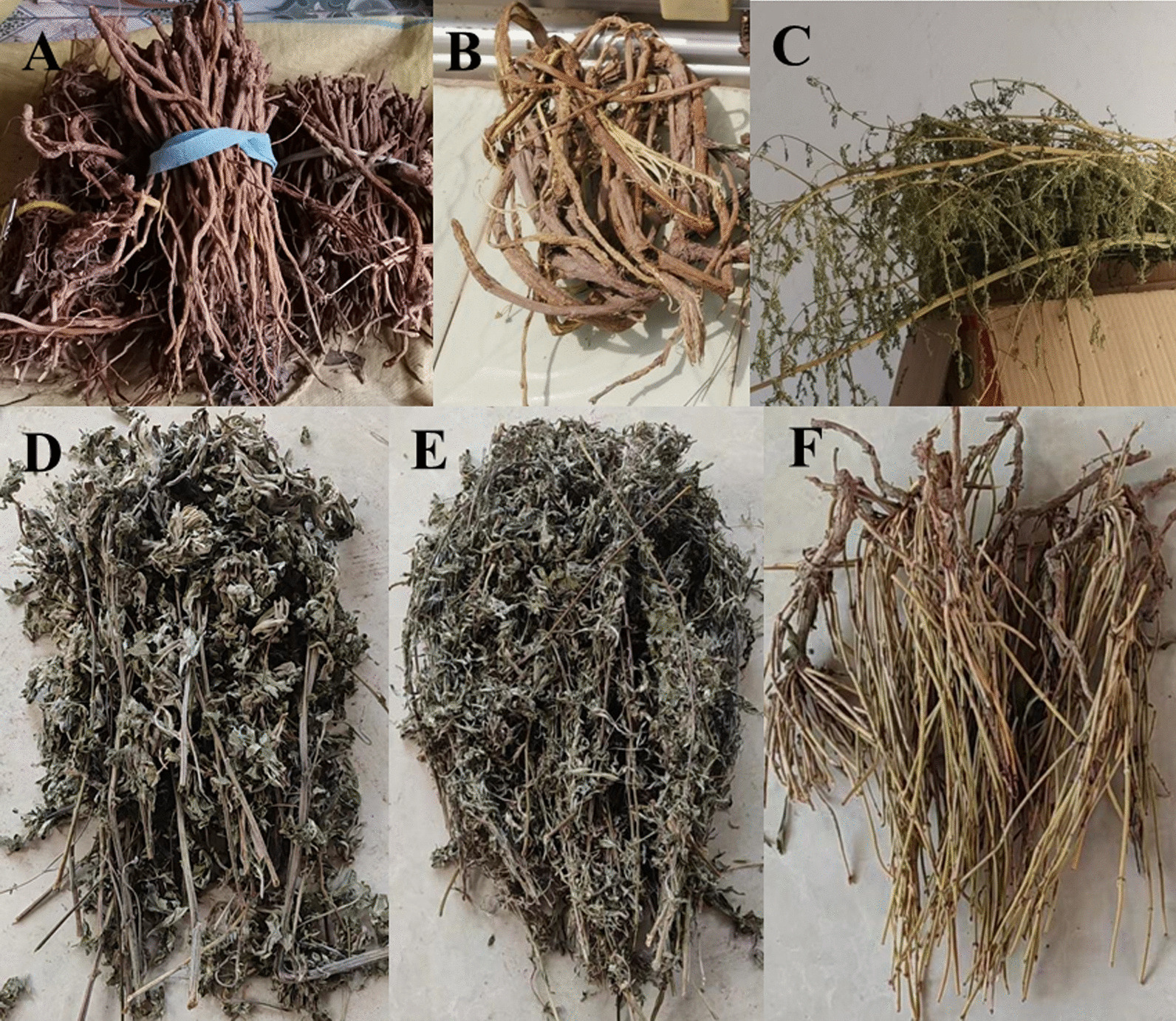
Common herbs stored by local residents. **A** *Scutellaria baicalensis* Georgi. (*S. baicalensis* Georgi.) **B** *Glycyrrhiza uralensis* Fisch. (*G. uralensis* Fisch.) **C** *A. annua* Linn. **D** A*. argyi* Lévl. et Van. (shui Ai). **E** *A. kanashiro*i Kitam. (Han Ai). **F** *E. sinica* Stapf

Commonly used medicinal herbs in households include *Artemisiae Annuae* Herba(*A. Annuae* Herba), *Artemisiae Argyi* Folium (*A. Argyi* Folium), *Bupleuri* Radix (*B.* Radix), *Glycyrrhizae* Radix et Rhizoma (*G.* Radix et Rhizoma), and *Ephedrae* Herba (*E.* Herba) (Fig. 3). These plants are mainly used as whole plant, with 15 species, followed by roots (14 species), stems (8 species), fruits and seeds (7 species), leaves (4 species), and bark (2 species). One particularly unique plant, *Euphorbia esula* Linn. (*E. esula* Linn.), is used for its fresh stem juice that oozes from the cut section. However, we did not collect information on wild flower herbs in the area, as local residents use cultivated flower herbs, such as safflower, chrysanthemum, and roses (mainly for consumption).

**Fig. 4:**
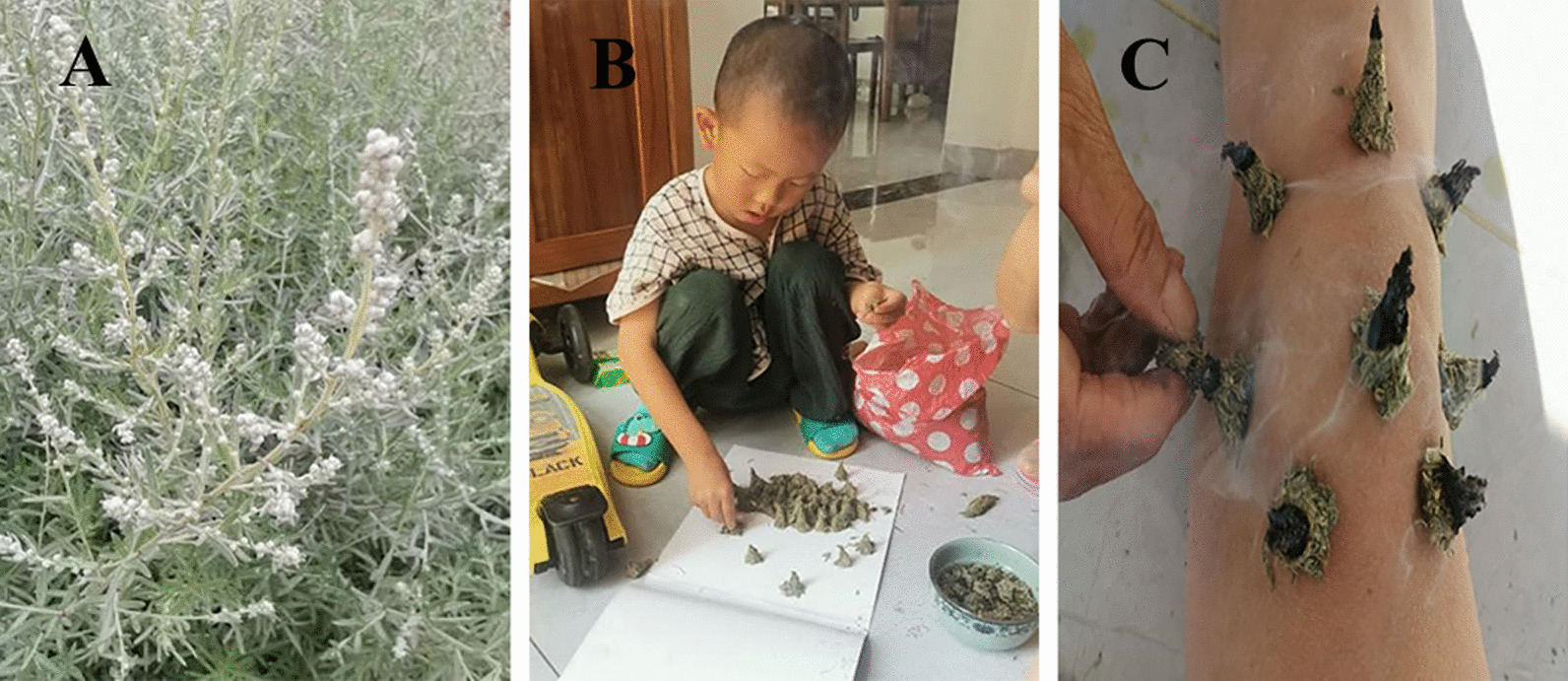
Moxibustion methods used by local residents. **A** Wild *A. kanashiro*i Kitam. **B** Local residents are making moxa sticks. **C** Local residents are using moxibustion to treat joint pain

The processing methods mainly involve direct sun-drying and cutting, with 35 medicinal plants using this processing method, followed by shade-drying (11 species), fresh use (7 species), and others (3 species). Local residents believe that sun-drying can cause the medicinal odor to disappear, thereby reducing or invalidating the therapeutic effect of the herbs. Therefore, herbs with fragrance are generally shade-dried. In addition, there are some special processing methods, such as the use of *A. argyi* Lévl. et Van./*A. kanashiroi* Kitam., which is generally pounded and rubbed into small mountain-shaped moxibustion bars. When used, it is dipped in saliva or water and directly attached to the skin surface using a burning incense stick (Fig. 4). It is removed when the skin feels hot, which is different from the general method used in traditional Chinese moxibustion therapy. Other medicinal herbs used for treating injuries and skin diseases are generally crushed and applied topically with fresh ingredients.

Local residents in the multi-ethnic regions where Gansu, Ningxia, and Inner Mongolia intersect use 50 medicinal plants, but only 27 of them are recorded in the Chinese Pharmacopoeia. Some medicinal plants used by local residents are also recorded in the Chinese Pharmacopoeia, but the sources are different. For example, the local residents use *Rhei* Radix et Rhizoma (*R.* Radix et Rhizoma), whose source plants are *R. undulatum* Linn. and *Rheum franzenbachii* Munt.(*R. franzenbachii* Munt.), while *B.* Radix mainly used by local residents is *B. smithii* Wolff var. *parvifolium* Shan et Y.Li, locally known as Xiao-CaiHu. In addition, some of the 50 wild medicinal plants used by local residents are also commonly used as wild vegetables, such as *T. mongolicum* Hand.-Mazz., *Sonchus arvensis* Linn. (*S. arvensis* Linn.), *Mulgedium tataricum* (Linn.) DC. (*M. tataricum* (Linn.) DC.), etc. [8].

### Quantitative evaluation of wild medicinal plants in the Hassan Mountain area

**Fig. 5:**
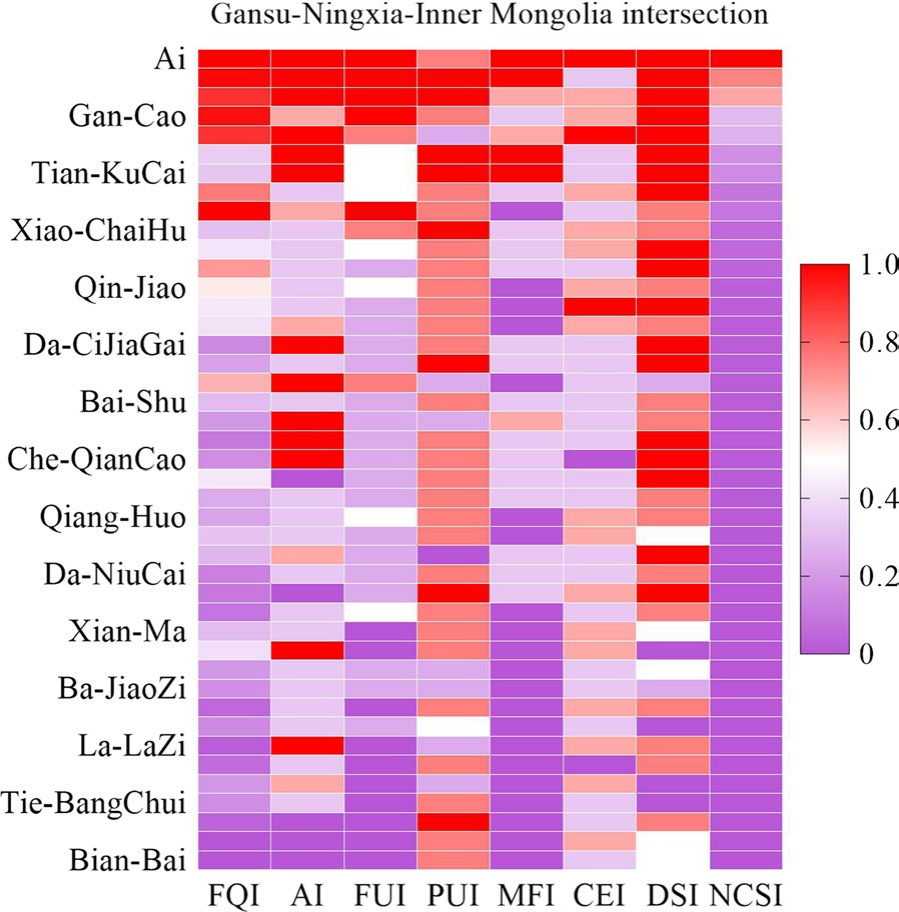
Heatmap of medicinal plants in Gansu–Ningxia–Inner Mongolia intersection zone

We quantified the importance of 44 wild herbs traditionally used by local residents in the Hasa Mountain area. The comparison results of the national plant cultural significance index (NCSI) of wild herbs in the region are shown in Table 3 and Fig. 5. Based on the NCSI, we clustered the wild herbs in the area, screened out wild herbs that are widely used, have high value, and play an important role in traditional healthcare among local people. The first important sequence (NCSI > 500) of herbs includes 9 species, representing plants such as Ai (including *A. argyi* Lévl. et Van and *A. kanashiroi* Kitam.), Huang-Hao (*Artemisia carvifolia* Buch.-Ham. ex Roxb. (*A. carvifolia* Buch.-Ham. ex Roxb.)), Huang-ErCai (*T. mongolicum* Hand.-Mazz.), Gan-Cao (*G. uralensis* Fisch.), Da-Yun (*C. tubulosa* (Schenk) Wight, *Boschniakia rossica* (Chamisso et Schlechtendal) B. Fedtschenko (*B. rossica* (Chamisso et Schlechtendal) B. Fedtschenko), etc. The first sequence of wild herbs plays an important role in the lives of local people in the area. They are natural medicines traditionally used by local residents to prevent and treat colds. These herbal resources have a wide distribution, are relatively easy to obtain, have high safety, and are mostly medicinal and edible plants, which are commonly used household items among local residents. The second important sequence (500 > NCSI ≥ 100) of wild herbs includes 15 species, representing plants such as Xiao-CaiHu (*B. smithii* Wolff var. *parvifolium* Shan et Y.Li), Huang-Qi (*Astragalus chrysopterus* Bunge (*A. chrysopterus* Bunge)), Suo-Yang (*C. songaricum* Rupr.), Qin-Jiao (*G. dahurica* Fisch.), etc. The third important sequence (100 > NCSI ≥ 10) of wild herbs includes 9 species, which are mainly plants with special distribution areas or toxic plants, such as Da-Huang (*R. undulatum* Linn.) of Qiang-Huo (*N. forbesii* de Boiss.). The fourth important sequence (10 > NCSI) of wild herbs mainly consists of toxic plants that are rare and less commonly used, such as Tian-XianZi (*H. niger* Linn.) and Tie-BangChui (*Aconitum brachypodum* Diels. (*A. brachypodum* Diels.)), etc.

**Table 3:** Quantitative evaluation index of wild herbs in the multi-ethnic areas of Gansu–Ningxia–Inner Mongolia intersection

Species	FQI	AI	FUI	PUI	MFI	CEI	DSI	NCSI
Ai	102	4	5	4	4	5	5	8,160.0
Huang-ErCai	101	4	5	5	4	3	5	6,060.0
Huang-Hao	92	4	5	5	3	4	5	5,520.0
Gan-Cao	99	3	5	4	2	4	5	2,376.0
Gou-Ci	92	4	4	2	3	5	5	2,208.0
Ma-KuMai	37	4	3	5	4	3	5	1,332.0
Tian-KuCai	35	4	3	5	4	3	5	1,260.0
Da-Yun	78	2	3	4	2	4	5	748.8
Ma-Huang	102	3	5	4	1	3	4	734.4
Xiao-ChaiHu	33	2	4	5	2	4	4	422.4
Huang-Qi	44	2	3	4	2	4	5	422.4
San-DanHua	72	2	2	4	2	3	5	345.6
Qin-Jiao	56	2	3	4	1	4	4	215.0
Suo-Yang	45	2	2	4	1	5	5	180.0
Zi-Ru	43	3	2	4	1	4	4	165.1
Da-CiJiaGai	17	4	2	4	2	3	5	163.2
Di-Jiao	24	2	2	5	2	3	5	144.0
Xing-He	67	4	4	2	1	3	2	128.6
Bai-Shu	32	2	2	4	2	3	4	122.9
Qian-SaoZhou	21	4	2	2	3	3	4	121.0
Ci-JiaGai	12	4	2	4	2	3	5	115.2
Che-QianCao	18	4	2	4	2	2	5	115.2
Yin-Chen	45	1	2	4	2	3	5	108.0
Di-Bai	27	2	2	4	2	3	4	103.7
Qiang-Huo	25	2	3	4	1	4	4	96.0
Da-Huang	34	2	2	4	1	4	3	65.3
Ma-ZiYou	30	3	2	1	2	3	5	54.0
Da-NiuCai	14	2	2	4	2	3	4	53.8
Ban-LanGen	12	1	2	5	2	4	5	48.0
Huang-Qin	11	2	3	4	1	3	4	31.7
Xian-Ma	32	2	1	4	1	4	3	30.7
Gou-ZhuaZi	42	4	1	4	1	4	1	26.9
Cang-Er	21	2	2	2	1	3	3	15.1
Ba-JiaoZi	19	2	2	2	1	3	2	9.1
Huang-XuanCi	7	2	1	4	1	4	4	9.0
Mao-ErYan	17	2	2	3	1	3	1	6.1
La-LaZi	4	4	1	2	1	4	4	5.1
Gui-Liu	8	2	1	4	1	2	4	5.1
Tian-XianZi	21	3	1	2	1	4	1	5.0
Tie-BangChui	18	2	1	4	1	3	1	4.3
Ma-PiPao	6	1	1	5	1	3	4	3.6
Xin-BuGan	2	1	1	4	1	4	3	1.0
Bian-Bai	2	1	1	4	1	3	3	0.7

## Discussion

In China, especially in rural areas, people have a wide-ranging tradition of using plants for disease prevention and treatment, which is influenced by traditional Chinese medicine culture. During the formation process of this tradition, rich local knowledge was generated [35]. In our investigation of the traditional knowledge of wild plant resource utilization in the multi-ethnic areas where Gansu, Inner Mongolia, and Ningxia converge, and a lot of important information about ethnobotany was discovered.

### Characteristics of wild medicinal plant resources

We found that the area has a rich diversity of wild medicinal plant resources, but only a small number of species are traditionally used by local residents, with a utilization rate of only 1/4. This result is closely related to the region's unique climate and ecological environment. A large portion of the 204 wild medicinal plant species we investigated are only found in the special environment of the Has Mountain Nature Reserve [36–38]. In fact, there are not many species that can be widely distributed in the area. At the same time, we found a phenomenon during our investigation: local residents often mention that "there are forty to fifty kinds of herbs here," which is consistent with the number of wild herbs we recorded that are used locally (44 species of herbs, 50 species of medicinal plants). This shows that the local residents' knowledge of wild medicinal plant resources in the area is limited to the species they use.

### Comparison with other ethnic groups in China

We compared the traditional Chinese medicine and medicinal plants used by other ethnic groups with those used by local residents in this area (in terms of species and usage methods). We found that only 27 of the herbs used by local residents are included in the Chinese Pharmacopoeia, and they have unique uses and methods. Overall, these herbs are closer to traditional Mongolian and Hui medicine, while they differ significantly from Tibetan, Miao, Gelao, Buyi, and Zhuang medicine [7, 39, 40]. This pattern is directly related to the geographical distribution of different ethnic groups in China. The main distribution areas of Mongolian and Hui people are adjacent to the region we investigated and have a certain intersection, with high consistency in terms of climate, plant resource distribution, etc. In contrast, the geographical distribution of Tibetans is special, and their traditional medicinal plants are mainly high-altitude plants. The Miao, Gelao, Zhuang, and Shui people mainly live south of the Yangtze River, with distinct differences in climate, plant resources, and living conditions compared to those in the northern arid and semi-arid regions. Therefore, it can be inferred that there is a direct correlation between traditional knowledge of herb usage and the environment among different ethnic groups and regions.

### Traditional knowledge of local residents on the utilization of wild medicinal plants

The local residents are most familiar with medicines for treating colds and rheumatism, as well as medicines for health preservation. The traditional use of medicinal plants in this region is mainly for treating colds, which is the most common disease, and the unique plant resources of this area are fully utilized. *E.* Herba (Ma Huang), as a controlled drug in China, is used more frequently by the residents of this area compared to other regions and is widely used to treat various colds. The most representative prescription for treating colds is the combination of *E.* Herba (Ma Huang), *A. Annuae* Herba (Qing Hao), and *Taraxaci* Herba (*T.* Herba) (Pu Gong Ying). Information on drugs used to treat cancer (*S. arvensis* Linn. and *M. tataricum* (Linn.) DC. is provided by people aged 40 to 60, most of whom have personal or family experiences of using them.

The most influential herb in the multi-ethnic and mixed wild medicinal plant resources of the Gansu–Ningxia–Inner Mongolia junction area is *A. Argyi* Folium, which is used as a medicine to treat various diseases in almost all age groups, especially for pain and gynecological diseases, and can be treated or alleviated with moxibustion (fumigation). However, the *A. Argyi* Folium used by local residents is significantly different from the variety used in the "Chinese Pharmacopoeia"[41]. Local people classify the local Artemisia plant into two categories according to their leaf shapes, and the *A. argyi* Folium (original plant: *A. argyi* Lévl. et Van.) included in the "Chinese Pharmacopoeia" is called "wide-leaved" or "round-leaved" and is known as "Shui-Ai" (meaning: Artemisia grown in water-rich areas or with high water content). The Artemisia plant used by local people is *A. kanashiroi* Kitam., known as "thin-leaved" and called "Han-Ai" (meaning: Artemisia grown in dry areas). Local residents firmly believe that the thin-leaved drought Artemisia they use is the best quality Artemisia, and that the wide-leaved water Artemisia has "insufficient fragrance and inadequate heat," and its moxibustion effect is far inferior to that of drought Artemisia [42]. This view is also reflected in "Huang-Qi". Local residents believe that the Tu-HuangQi (locally produced *Astragali* Radix (*A.* Radix)) they use is more effective than *A. membranaceus* (Fisch.) Bge. and Astragalus membranaceus (Fisch.) Bge. var. mongholicus (Bge.) Hsiao (*A. membranaceus* (Fisch.) Bge. var. *mongholicus* (Bge.) Hsiao), but they believe that the differences in the efficacy of Huang-Qi mainly come from differences in cultivation, wild or growing age, rather than differences in species. In addition, local residents' use of moxibustion methods differs from traditional Chinese medicine methods. They directly paste the moxa on the surface of the skin and remove it when they feel pain. *S. arvensis* Linn., *M. tataricum* (Linn.) DC., and *T. mongolicum* Hand.-Mazz. are not only used by local residents to treat diseases but are also primarily consumed as vegetables [43].

### Local residents' awareness of the conservation of wild medicinal plants

There was a period of time when the wild liquorice (*G. uralensis* Fisch) in the area suffered serious destruction (as locals put it: "20 years ago, liquorice almost went extinct in our area") [44]. However, in the past decade, almost no one has been digging wild liquorice, but instead, they have collected a large number of liquorice seeds. Although this behavior is mainly influenced by economic income, it has played a very important role in the conservation of wild liquorice [45]. The area is arid and has little rain, and liquorice seeds have almost no chance of sprouting in their natural state (wild resources mainly propagate through the root system), but a large number of high-quality liquorice seedlings can be formed through artificial breeding for agricultural production. At the same time, large-scale planting of Ningxia wolfberry (*L. barbarum Linn.*) [8] in the area has provided ample job opportunities for local residents, which is also an important factor in avoiding large-scale harvesting of wild medicinal plants. In addition, the traditional Chinese medicine *Notopterygium incisum* Ting ex H. T. Chang (*N. incisum* Ting ex H. T. Chang), *Gentiana macrophylla* Pall. (*G. macrophylla* Pall.) and *Bupleurum chinense* DC. (*B. chinense* DC.) were widely planted and managed, which has effectively promoted the conservation and sustainable use of the wild medicinal plant resources in the area [29].

Overall, the traditional knowledge and practices of local residents in the multi-ethnic region of Gansu, Ningxia, and Inner Mongolia regarding the use and conservation of wild medicinal plants are unique and valuable. It is essential to recognize and respect the cultural diversity and traditional knowledge of local residents, and to integrate their knowledge and practices into the conservation and sustainable use of wild medicinal plants. This will contribute to the preservation of biodiversity, the promotion of sustainable development, and the improvement of the health and well-being of local communities.

### Current use of traditional knowledge of wild medicinal plants by local residents

In China, many traditional knowledge and cultural heritage are disappearing with the intensification of economic development and urbanization [46, 47]. The traditional knowledge of local residents in using wild medicinal plant resources is also facing this fate. Many traditional cultures that can be preserved and continued are mostly passed down in the form of written records or museums [48–50], but there are few written records of traditional knowledge about local wild plants used for medicinal purposes.

Most of the valuable information we obtained in the survey was provided by respondents over 40 years old, especially elderly village doctors who provided the most abundant information. Young people were able to provide very little valuable information, and traditional village doctors were also facing the dilemma of having no one to inherit their knowledge. Local traditional knowledge about medicinal plants is gradually disappearing, and there may be several reasons for this. Traditional herbal medicine mainly targets symptoms and focuses on alleviating patients' discomfort rather than addressing the root cause of the disease, and the range of diseases they can treat is very narrow. Compared with modern medicine and traditional Chinese medicine, local herbal doctors have greater variability in treating diseases, and a considerable number of herbal doctors do not use measuring tools when preparing herbal remedies. The overall sanitation conditions in herbal doctors' clinics are also deviating, which may make it difficult to ensure the uniformity of the efficacy of their herbal remedies. In addition, with the development of the local economy and transportation, people are more willing to go to large hospitals for treatment and to trust in traditional Chinese medicine or modern medicine.

## Conclusion

In the study of wild medicinal plant resources in the multi-ethnic areas of the Gansu–Ningxia–Inner Mongolia convergence zone, it was found that the region has abundant wild medicinal plant resources, but local residents only use a small amount of the species, which is related to the unique climate and ecological environment of the region. Local residents' knowledge of wild medicinal plants is limited to the species they use, especially in the treatment of colds and rheumatism, where traditional knowledge is fully utilized, and some herbs have very unique uses and methods. These herbs are closer to traditional Mongolian and Hui medicine, rather than drugs used by Tibetan, Miao, Gelao, Buyi, and Zhuang ethnic groups. However, this traditional knowledge is facing a huge risk of disappearing. Our work not only preserves the vitality of traditional knowledge but also provides first-hand information for in-depth research and development of these special herbs.

## Supplementary Information

**Additional file 1.** Ethnobotanical research questionnaire.

## Data Availability

All data, materials, and information are collected from the study sites.

## Abbreviations

NCSI: Cultural food significance index
FQI: Frequency of quotation index
AI: Availability index
FUI: Frequency of utilization index
PUI: Parts used index
MFI: Multifunctional use index
CEI: Curative effect index
DSI: Drug safety index
